# Will Artificial Intelligence Be “Better” Than Humans in the Management of Syncope?

**DOI:** 10.1016/j.jacadv.2024.101072

**Published:** 2024-07-31

**Authors:** Franca Dipaola, Milena A. Gebska, Mauro Gatti, Alessandro Giaj Levra, William H. Parker, Roberto Menè, Sangil Lee, Giorgio Costantino, E. John Barsotti, Dana Shiffer, Samuel L. Johnston, Richard Sutton, Brian Olshansky, Raffaello Furlan

**Affiliations:** a Internal Medicine, IRCCS Humanitas Research Hospital, Rozzano, Italy; b Division of Cardiovascular Medicine, Roy J. and Lucille A. Carver College of Medicine, University of Iowa, Iowa City, Iowa, USA; c IBM Technology Expert Labs, Milan, Italy; d Department of Biomedical Sciences, Humanitas University, Milan, Italy; e Cardiac Arrhythmia Department, Bordeaux University Hospital, INSERM, Bordeaux, France; f IHU LIRYC, Electrophysiology and Heart Modeling Institute, Bordeaux, France; g Department of Emergency Medicine, Roy J. and Lucille A. Carver College of Medicine, University of Iowa, Iowa City, Iowa, USA; h Emergency Department, IRCCS Ca’ Granda, Ospedale Maggiore, Milano, Italy; i Department of Epidemiology, College of Public Health, University of Iowa, Iowa City, Iowa, USA; j Department of Cardiology, Hammersmith Hospital Campus, National Heart & Lung Institute, Imperial College, London, United Kingdom

**Keywords:** artificial intelligence, clinical decision, education, research, syncope

## Abstract

Clinical decision-making regarding syncope poses challenges, with risk of physician error due to the elusive nature of syncope pathophysiology, diverse presentations, heterogeneity of risk factors, and limited therapeutic options. Artificial intelligence (AI)-based techniques, including machine learning (ML), deep learning (DL), and natural language processing (NLP), can uncover hidden and nonlinear connections among syncope risk factors, disease features, and clinical outcomes. ML, DL, and NLP models can analyze vast amounts of data effectively and assist physicians to help distinguish true syncope from other types of transient loss of consciousness. Additionally, short-term adverse events and length of hospital stay can be predicted by these models. In syncope research, AI-based models shift the focus from causality to correlation analysis between entities. This prompts the search for patterns rather than defining a hypothesis to be tested a priori. Furthermore, education of students, doctors, and health care providers engaged in continuing medical education may benefit from clinical cases of syncope interacting with NLP-based virtual patient simulators. Education may be of benefit to patients. This article explores potential strengths, weaknesses, and proposed solutions associated with utilization of ML and DL in syncope diagnosis and management. Three main topics regarding syncope are addressed: 1) clinical decision-making; 2) clinical research; and 3) education. Within each domain, we question whether “AI will be better than humans,” seeking evidence to support our objective inquiry.

## Highlights

• Syncope management creates challenges for patients and their families, clinicians, learners, and institutions—AI may enhance management.
• Strengths/weaknesses of AI in syncope care are assessed in clinical decision-making, research, and education.
• AI will likely be able to distinguish syncope from other causes of loss or alteration of consciousness.
• A key feature of AI is that it can define new patterns to help achieve expected goals of improved syncope management.

In medicine, artificial intelligence (AI), particularly machine learning (ML) and deep learning (DL)-based techniques along with natural language processing (NLP), can efficiently analyze vast amounts of data. AI-based approaches integrate diverse multimodal data inputs, reveal unconventional associations, and identify unique connections between risks and diseases.^1^^,^^2^ Consequently, potential to enhance the accuracy of diagnosing and managing illnesses exists.^3^ Such complex tasks can overwhelm clinicians. Moreover, ML, DL, and NLP models show promise in estimating hospital length of stay,^4^ and assisting policymakers to optimize health care costs. These advances are particularly relevant to manage syncope.^1^ However, limitations, weaknesses, and ethical concerns exist.^5^^,^^6^

Here, we explore the provocative issue whether AI techniques can surpass human capabilities, with specific focus on syncope diagnosis and management (Central Illustration). Our objective is to highlight potential strengths, weaknesses, and solutions related to use of NLP, ML, and DL. We will address 3 principal syncope topics: 1) clinical decision-making; 2) clinical research; and 3) education. We will consider ethical aspects that may arise from use of ML, DL, and NLP models.

**Central Illustration:**
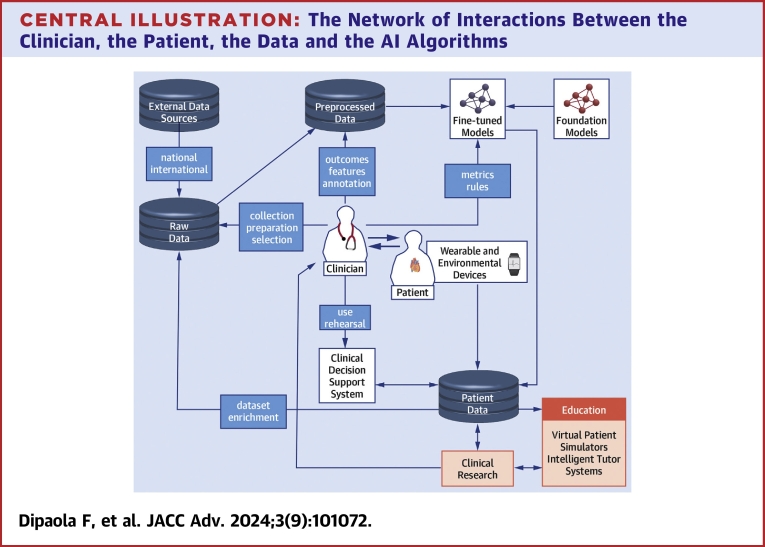
**The Network of Interactions Between the Clinician, the Patient, the Data and the AI Algorithms** Raw quality data sets are the starting point of the clinical decision support system (CDSS). National and international data may include time series obtained from wearable and environmental devices. preprocessed and properly “formatted” data may be used to train ML/DL models directly or by fine-tuning a pretrained model (eg, a foundation model). CDSS developed based on these models can be used to test and create systems that can assist the clinician with diagnosis, prognosis, education, and patient’s clinical management. Feedback between clinicians and CDSS can help generate new data for the system. The entire cycle is then repeated (data set enrichment). The same data can be used for educational purposes (eg, virtual patient simulators) and clinical research. AI = artificial intelligence; DL = deep learning; ML = machine learning.

### AI will be “better” than physicians in syncope clinical decision-making

Studies comparing performance of ML to humans regarding syncope are lacking. However, recent evidence on applications of ML and large language models in clinical reasoning and decision-making^7^ suggest advantages of these technologies for syncope management. Two distinct human-AI interactions exist: 1) human decisions with support from AI-based software, as seen with clinical decision support system (CDSS); and 2) AI software decisions made independently, that incorporate real-time dynamic data sourced from available tools, such as wearables. CDSS software is designed to assist clinicians’ decision-making by a computerized knowledge data set to generate personalized assessments or recommendations based on patient characteristics. CDSSs are used primarily at point-of-care, integrated with electronic medical records (EMRs)/electronic health records (EHRs) or through web applications, allowing clinicians to combine their expertise with information provided by the CDSS. CDSSs can enhance clinicians' capabilities by minimizing prescribing errors, promoting guideline adherence, enabling treatment reminders, cutting health care costs (eg, minimizing test and order duplication), and offering diagnostic help by analyzing patient data and interpreting test results. Moreover, CDSSs can improve existing workflows.^8^

Recently, there has been a trend toward developing CDSSs that leverage AI techniques, such as ML and statistical pattern recognition, to analyze complex data and observations; this might be challenging for humans to interpret but may be critical to arrive at a management plan for patients presenting with syncope.^8^ These advanced CDSSs, known as non-knowledge-based, require a data source, but the decision-making process relies on ML or learning a statistical pattern from available data rather than following predefined rules. This is a major advantage over conventional rule-based models because a model with its own decision-making process can learn new methods of recognition over time. Such an approach may augment present human-based endeavors to optimize the individual approach to the patient with syncope. CDSS powered by AI chatbots^6^ could be incorporated into EMR/EHR platforms to provide differential diagnoses and preliminary treatment plans for patients with syncope. Recent studies evaluating AI chatbots’ responses to sample clinical cases show promising results.^9^^,^^10^

Variability in syncope symptom presentation, origin, end points, and risk factors, combined with absence of a specific diagnostic test and limited array of effective treatment options, complicates clinical decision-making and increases potential for physician errors.^11^, ^12^, ^13^, ^14^, ^15^ A correct etiological syncope diagnosis is crucial to determine accurate diagnostic and appropriate therapeutic pathways.^13^^,^^14^ It is essential during the diagnostic phase to explore only relevant secondary investigations, ensuring resources are used efficiently to minimize patient risks related to unnecessary hospitalizations and over-testing that may create harm.^16^ Moreover, clinicians should choose the most suitable treatment based on the etiology of the syncope. If syncope remains undiagnosed after the initial evaluation (ie, history, physical examination, ECG), it is imperative to undertake thorough risk assessment to promptly identify patients at high risk who might benefit from urgent treatment.^17^

While several risk stratification tools and score systems have been developed, they have only partially achieved effective risk stratification. Notably, no studies compare different syncope prognostic tools (SPTs) directly, leaving a gap in knowledge regarding the best recommendations. Few SPTs have undergone external validation.^18^, ^19^, ^20^ Since syncope-related morbidity and mortality is uncommon, limited size derivation studies are unlikely to include such patients, impacting SPT sensitivity when attempting validation. Furthermore, different SPTs focus on varying outcomes, for example, short- versus long-term adverse events. Existing syncope decision rules have not consistently demonstrated increased diagnostic accuracy^18^ or cost savings.^19^ Finally, performance of SPTs was not found superior to clinical judgment in syncope risk stratification.^21^ Therefore, there is need to properly address syncope’s complex and interrelated features. ML, DL, and NLP might accomplish these aims by revealing hidden associations or unexplored patterns, thus improving diagnostic accuracy, risk stratification, interpretation of laboratory or radiological tests, and personalized treatment compared to physicians.

A few preliminary studies proposed knowledge-based CDSSs to apply clinical guidelines, or risk scores^22^ that are hoped to be applicable in managing syncope in emergency settings.^20^ AI-driven, non-knowledge-based CDSSs to determine their capability are anticipated. However, the impact of CDSSs, knowledge-based or otherwise, on patient outcomes and treatment pathway optimization is yet to be demonstrated due to limited implementation in clinical practice, and lack of external validation.^23^ Thus, no data demonstrate that AI is “better” than physicians at syncope clinical decision-making yet.

In outpatient settings, AI has been extensively used to analyze data from noninvasive wearable devices (eg smartwatches, fitness bands, glasses) that monitor physical status and physiological parameters continuously.^24^^,^^25^ Wearable device use has surged to >70 million U.S. adults.^26^ Despite ongoing concerns for diagnostic accuracy,^24^ wearables, when integrated with AI, might identify underlying pathophysiological mechanisms such as the sudden onset of atrial fibrillation with high rate ventricular response,^27^ or malignant arrhythmias responsible for syncope and prompt personalized medical interventions. Groppelli et al developed a wearable device that can be activated during prodromal symptoms. Additionally, this device can detect blood pressure variations preceding vasovagal syncope using tilt-testing.^2^^,^^28^ Some implanted monitors can detect position and activity at the time of collapse. Emerging technologies can detect fall or alert patients about their blood glucose levels.^29^ No doubt, capabilities of wearables are increasing rapidly to provide further diagnostic acumen regarding episodes of collapse.

Current AI-based algorithms, known as “symptom checkers,” can assist patients with self-diagnosis or self-triage when they experience symptoms,^30^ including syncope. Importantly, by correlating reported symptoms with real-time physiological changes detected by wearables, most syncope episodes, particularly low-risk ones, could be easily identified and automatically transmitted to their general practitioners for clinical evaluation, avoiding unnecessary emergency department visits, and need for specialized consultation. Thus, AI models might influence overall costs associated with syncope management positively. These algorithms should maximize sensitivity if they aim to identify low-risk syncope patients.

Additionally, AI’s ability to analyze vast amounts of data might enable monitoring of large outpatient populations, possibly preemptively, identifying high-risk profiles among patients suffering from syncope, for example, individuals with pre-excitation, Brugada pattern, long QT, hypertrophic cardiomyopathy, and arrhythmogenic right ventricular cardiomyopathy.^31^ However, emerging wearable technology with AI techniques has limitations (Table 1). The reliability of outcomes largely depends on quality of data input into ML or DL algorithms. In real-world scenarios, movement-related artifacts can degrade the quality of recorded signals, potentially undermining the reliability of AI analyses. As such, AI may hinder delivery of proper care. Additionally, ectopy and non-perfusing rhythms can be misclassified by photoplethysmography as bradycardia.^32^ Therefore, we are not yet ready to make clinical decisions solely on current wearable technology. Data produced by wearables are sensitive and likely to require specific legal regulations to ensure patient privacy, leading to limited integration with EMRs/EHRs. Moreover, distribution of wearables is heterogeneous within the population, possibly exacerbating disparities.^26^ Despite this, ML, DL, and NLP models, and AI-based chatbots have shown promise to analyze vast amounts of data^3^^,^^32^ to help distinguish benign from life-threatening causes of syncope,^23^ as well as distinguish syncope from other forms of transient loss of consciousness.^2^^,^^33^ Additionally, short-term adverse events^23^^,^^34^, ^35^, ^36^ and length of hospital stay^4^^,^^37^ proved to be properly predicted by AI-based models. This may facilitate establishment of standardized protocols for syncope management and personalized treatments.

**Table 1:** Benefits of AI Systems Use in Syncope Clinical Decision-Making, Potential Harms, and Proposed Solutions

Strengths	Weaknesses	Proposed Solutions
Uncover unconventional disease associations	Performance hindered by low-quality data and documentation, or high disease complexity	Create datasheets as a quality requirement for each data set
Expedite electronic data gathering	May reinforce patient biases	Use a data statement to educate NLP developers about the software
Standardize protocols for syncope management	No legal regulations of liability and patient privacy	Employ medical experts to improve data labels in retrospective data sets
Identify rare etiologies; ensure common etiologies are not overlooked	Can provide predictions without clear reasoning—“black boxes”	Implement self-supervised AI to improve data labeling
Predict readmission risk and prevent unnecessary hospitalization	Reliance on AI could reduce medical professional critical thinking	Utilize small data sets to focus on specific populations and rare conditions
Minimize unnecessary testing by enhancing guideline adherence	High expense, thus inaccessible to medical groups without adequate fundings	Develop AI models, such as hybrid architecture models, that provide transparent and interpretable results
Identify real-time physiological changes before the loss of consciousness	May fail to understand patient preferences; recommendations may not align with patient values	Encourage collaboration among software developers, clinicians, and researchers to validate AI application
Identify high-risk patients	Physical activities reduce wearable monitor data quality	Establish ethical frameworks to address biases and discrimination in AI predictions
Allow for personalized, adjusted therapies		Validation studies of data from wearables
Might be one means of alleviating the inequality between urban and rural health services in developing countries		

AI = artificial intelligence; NLP = natural language processing.

A major limitation of the clinical effectiveness of any AI-based model is the quality of data they rely upon.^5^ That concept applies to syncope too.^23^^,^^34^, ^35^, ^36^ Practical suggestions to improve syncope data quality are summarized in the third column of Table 1. Several actions have been hypothesized to accomplish that aim, including creating datasheets for each data set to be analyzed and ensuring efficient data labeling when necessary.^5^ Datasheets should document the motivation underlying dataset creation, composition, collection process, preprocessing/cleaning/labeling procedures, recommended uses, distribution, maintenance, potential impact, and updates.^5^ Similarly, data statements have been proposed to provide NLP developers and users with information regarding major features and limitations of the NLP software.^38^

To improve integration of AI in medical decision-making scenarios, the development of models that provide transparent and interpretable results^39^ is essential (see Table 1). This may help professionals better understand the reasoning behind AI recommendations and address concerns during critical and shared decision-making situations. Furthermore, transparency of these models may preserve patient autonomy and provide reassurance to those who are apprehensive of AI. Combination of multiple small data sets, the intrinsic quality of which is likely to be more efficiently controlled, might lead to more relevant and context-specific insights.^5^

Currently, within clinical processes, the ultimate decision is solely made by physicians. However, in other processes, AI plays a more expansive role. Decisions can be made in collaboration with AI and, in certain scenarios, by AI alone based on human-provided data. Emerging decision-making models require further evaluation. Ascertaining whether incorporation of AI yields tangible advantages for patients could propel its integration within medical contexts, potentially mitigating associated legal and ethical challenges. Thus, regarding syncope decision-making, AI does not yet outperform humans. However, it may be hypothesized that AI can assist physicians in syncope diagnosis and management. Future studies should provide relevant evidence. The focus remains on human intelligence, with AI algorithms serving as valuable tools, to augment medical professionals’ capabilities.

### AI will be “better” than researchers in syncope clinical research

Tremendous potential exists to integrate large amounts of data and evidence from known research to enhance syncope evaluation for an individual patient. Despite being a common clinical event, short-term adverse events related to syncope are infrequent. Thus, to include a significant number of these events in research studies, prospective enrollment of large patient cohorts becomes necessary, demanding substantial time and resources.

Attribute matching proved to be a valuable technique to personalize risk stratification in clinical settings characterized by nonspecific symptoms, including syncope.^40^ Similarly, AI and ML have potential to enhance and streamline clinical research by facilitating more efficient recruitment and matching of study participants and enabling comprehensive data analysis.^41^

EMRs/EHRs provide essential information for database development although, prospectively and systematically gathered data sets may furnish other important advantages concerning the data quality. Presently, information is generally stored as unstructured data, necessitating extensive human intervention for interpretation. The combined analysis of structured and unstructured data by NLP techniques may facilitate prompt identification of data from patients with syncope from EMRs/EHRs and administrative repositories.^42^

Moreover, AI has the ability to capture nonlinear correlations, to combine diverse data types and to operate without a priori assumptions, frequently outperforming traditional classical statistical methods. In research settings, AI chatbots can help researchers formulate questions, develop study protocols, and summarize data.^22^^,^^41^ Algorithms can provide researchers with autonomously generated notes and reports thus potentially acting as a dialectical companion, fostering formulation of novel research hypotheses and objectives.

Only a few observational studies^23^^,^^34^^,^^35^^,^^42^ have analyzed application of ML to syncope detection and risk prediction. Despite encouraging preliminary results, these models lack external validation in clinical contexts beyond the ones used for their development. Notably, external validation is crucial to demonstrate generalizability and practical usefulness of these models in clinical practice.

Large language models can extract data from EMRs/EHRs, enabling the analysis of both structured and unstructured data, with particular emphasis on syncope prognosis, management, and therapy (Table 2). AI chatbots can help develop protocols and manage data for syncope investigations. The use of AI-based models has brought about a shift in the previous epistemological paradigm, moving away from a focus on causality to a novel approach centered around correlation analysis between entities in 2 data sets (Table 2). This change has led to the search for patterns and an uptick in hypothesis-generating exercises.^5^ Rather, AI enables new hypothesis generation based on previously unseen patterns in data. These concepts are particularly relevant to syncope research.

**Table 2:** Benefits of AI Systems Use in Syncope Clinical Research, Potential Harms, and Proposed Solutions

Strengths	Weaknesses	Proposed Solutions
Assist in research protocol development and data summarization	Manual data selection made by inexperienced individuals	Syncope expert physician manual review to validate data
Aid participant recruitment	AI use requires technical skill integration between IT and medical researchers	Validate ML models in diverse clinical contexts with multicenter studies
Collect structured and unstructured data from EMRs/EHRs	ML interventions in clinical research are not standardized	Training programs combine IT and medical domains
Paradigms focused on correlation are not constrained by medical theory; may uncover unique associations	ML in syncope is poorly studied; current models lack external validation	AI experts, physicians, and researchers collaborate to develop robust ML models
Replace need for traditional statistical sample size estimation	High cost data sets	Small, focused data sets for improved data accuracy and more reliable predictions
Often outperform classical statistics like multivariable analysis	Inequitable access could exacerbate research disparities	Cost-effective and faster data sets can address privacy and ethical concerns
	Ethical concerns about owning large-scale patient data sets	Develop best practice guidelines
		Data sharing initiatives and collaboration between big and small research entities to promote equitable clinical research
		Policymakers and industry stakeholders create data-sharing and privacy frameworks

AI = artificial intelligence; EHRs = electronic health records; EMR = electronic medical record; ML = machine learning.

The lack of standards describing and testing ML interventions, and the absence of external validation represent major weaknesses of the published studies on syncope. Ensuring data accuracy and minimizing errors can be achieved through human oversight in the data selection process and manual review by medical professionals (Table 2). To establish effectiveness and generalizability of ML models for syncope detection and risk prediction, external validation studies through multicenter collaborations will be required.

Compared to large data sets, small data sets offer greater focus and careful curation, ensuring higher data quality potentially leading to more reliable predictions and outcomes. However, small data sets are sometimes used because no ‘large’ data sets are available with the required information. A form of AI, known as the generative adversarial network, can potentially rectify this lack of data, supplementing small data sets with synthetically generated patient-level data.

To promote equitable and ethical clinical research (Table 2), transparent data-sharing practices and collaborations between major institutions and smaller research entities are essential. In this context, AI and various models serve as valuable tools in support of humans, who remain at the core of clinical research.

### AI will be “better” than humans in syncope education

Building clinical and diagnostic reasoning abilities is integral to medical education for medical students and seasoned doctors alike, not to mention a lifelong learning journey. Moreover, for patients, understanding the cause of syncope enhances compliance, encourages nonpharmacological approaches, and improves overall quality-of-life, reducing anxiety about the event and future recurrences. Nevertheless, syncope is a complex clinical presentation with a wide spectrum of elusive etiologies and no singular diagnostic pathway or treatment strategy.^43^ Worldwide, syncope has not been adequately emphasized as part of medical school training. While curricula may vary between medical schools, medical students may be only briefly introduced to syncope as a presenting symptom in preclinical lectures, ECG interpretation, valve disease, or neurology. Yet, they are expected to manage patients with syncope on their first year of residency. Specialty training programs also underestimate syncope training in their curriculum. Recently, some institutions and nonprofit organizations, such as Syncopedia Foundation, started to offer e-learning modules to enhance learner knowledge of presumed syncope identification and reduce misdiagnosis, unnecessary testing, and excessive specialist consultations.^44^ AI may be able to address this major educational gap and help novice doctors deal with clinical cases. Indeed, teaching hospitals may spend more effort on the diagnostic assessment of syncope without improvement in patient outcomes compared to nonteaching hospitals.^45^ Furthermore, intensive syncope-focused education for hospital-based physicians may improve fundamentals, guideline adherence, and refine syncope risk stratification.^46^ No matter, student or physician, adequate training and coaching regarding syncope are pivotal to develop diagnostic skills that may improve cost-effective and patient-centered care.

Currently, in medical schools and residency programs, clinical coaching occurs under direct supervision of senior doctors, primarily within hospital wards. However, constraints in human resources, rising educational costs, and social distancing measures, imposed by the COVID-19 pandemic,^47^ highlight the need for training methods that do not solely rely on bedside teaching and direct supervision.^48^ The recent surge in enthusiasm for AI in medical education is aimed at refining the pedagogical methods for medical students and practicing physicians, to endow them with means to handle massive data loads, and ultimately enhance their abilities to formulate differential diagnoses that can consequently improve patient safety and reduce health care costs. One example is the use of virtual patient simulators to enhance clinical diagnostic reasoning^11^^,^^49^ that may aid in teaching students how to assess complex clinical presentations. These interactive teaching tools can build virtual patients using real patient data. They can adjust the complexity of scenarios to the learner’s competency level. Syncope, being a symptom underlying a wide range of clinical conditions with varying prognoses, represents a paradigmatic example of educational complexity. Collecting the clinical history is fundamental to the diagnostic process^13^^,^^14^ and consequently is crucial for syncope education.

One principal reason to employ AI in medical training is its potential to offer instant feedback, a key factor in identifying learning objectives and understanding deficits in medical students’ knowledge. Indeed, learners, aware of their lack of knowledge, may devise strategies for self-improvement. Intelligent tutoring systems have been designed to offer instant, tailored instruction and feedback without human intervention, showing effectiveness in various educational settings.^50^

The role of AI in medical education may not be limited to just medical trainees and physicians; patients also stand to benefit. NLP-based systems have been proposed to educate patients about their medical conditions and answer questions.^51^ This could greatly help patients who have a likelihood of syncope recurrence. For example, despite the often-benign nature of reflex syncope, patients report a reduced quality-of-life due, in part, to unanswered questions and poor understanding of their condition.^52^ NLP-driven patient education after syncope could learn from these questions and reasonably bridge gaps between office appointments to reduce patient uncertainty, improve quality-of-life, and alleviate anxiety related to syncope.

As outlined in Table 3, application and effectiveness of AI in medical education have challenges. To determine the success of an AI system, a scientific approach rooted in “explainability” is needed. This is especially critical in DL, a branch of AI, where, due to its complex, nonlinear nature, the rationale behind AI decisions may remain unclear.^53^ In medical education, understanding these decision-making processes is vital. For example, in vasovagal syncope, understanding the autonomic pathways involved, including triggers,^13^^,^^14^ helps direct questions as part of the clinical history. By contrast, algorithms behind how AI arrives at decisions can be unclear since they typically evolve based on training/retraining sets. Given the complexity of syncope, that decision-making process will require close monitoring and consistent training of data through accurate and effective feedback mechanisms.

**Table 3:** Benefits of AI Systems Use in Syncope Teaching and Education, Potential Harms, and Proposed Solutions

Strengths	Weaknesses	Proposed Solutions
Chatbots can supplement traditional educational resources	Chatbots can fabricate educational information that seems plausible (“hallucinations”) and make up references to support its fallacious responses	Humans validate chatbots’ educational outputs and confirm alignment with current evidence-based guidelines
AI can create instructive and interactive clinical vignettes	AI may fail to train empathy or strengthen effective communication	Refine existing AI models to recognize emotion and respond empathetically
AI can analyze students' performances, identify knowledge gaps, and implement remediation strategies	AI-based technology, especially visual reality, can be costly and require technical expertise not available in all educational settings and socioeconomic contexts	Physicians review syncope clinical vignettes
AI can provide real-time feedback	Rely on the veracity of training data sets to simulate clinical scenarios accurately and provide substantive feedback	AI experts, physicians, and teachers collaborate to develop robust AI models of specific syncope simulated cases
Application of adaptive learning into AI-powered platforms	Restricted to clinical knowledge that it has access to and was trained on	Likewise, collaboration to confirm AI models is effective in medical education
Instant access to updated credible resources, ie, clinical cases library	Affordances of using a specific type of AI-powered intelligent tutoring system to provide feedback may be limited by risks of inaccurate feedback, high cost, and lack of social interaction with teachers and peers	Educational grants at national and institutional level
Broad and free of language barrier application, available to those with learning disabilities	Depends on internet and network availability	Widely available internet especially in rural areas

AI = artificial intelligence.

Designing a syncope AI educational system requires a multidisciplinary team of syncope specialists, educational experts, and data scientists. There may be considerable challenges to match the rapidly evolving practices, innovations, and trends from AI/ML. Engineers and data scientists prioritizing prediction accuracy may not always align with clinical and educational relevance. Intelligent tutoring systems show promise in addressing educational gaps.^54^ However, they are inferior to human supervision, may lead to inaccurate feedback, and require financial support. Lack of social interaction with teachers and peers may adversely affect student attitude, which may further limit the implementation of these tools.

An NLP-based virtual patient simulator could offer students and doctors engaged in their continuing medical education the opportunity to challenge themselves with an unlimited number of lifelike syncope clinical cases. The interactive nature of NLP allows users to engage in realistic clinical scenarios, refining their history-taking abilities, diagnostic skills, and management capabilities.

Recently, AI-based dialogue systems called “chatbots” have been implemented in multiple domains of medical and patient education.^55^ Typically, a human starts a conversation by entering a query and the chatbot provides a prompt response through natural-language capability. However, as chatbots are trained on extensive open-access sources, they may provide inaccurate responses and fabricated information, referred to as “hallucinations.”^22^ This could jeopardize the learning experience for medical students and continued education for health care professionals. Furthermore, virtual patient simulators may be unable to adequately train empathy or effective interpersonal communication skills the way direct patient interaction can.^56^^,^^57^ Such skills are crucial for establishing a successful patient-physician relationship during syncope assessment. So far, AI cannot replace the “art” of medicine—the subjective, nuanced, and interpersonal component of medicine that trainees learn only from hands-on experience.

## Conclusions

**Figure 1:**
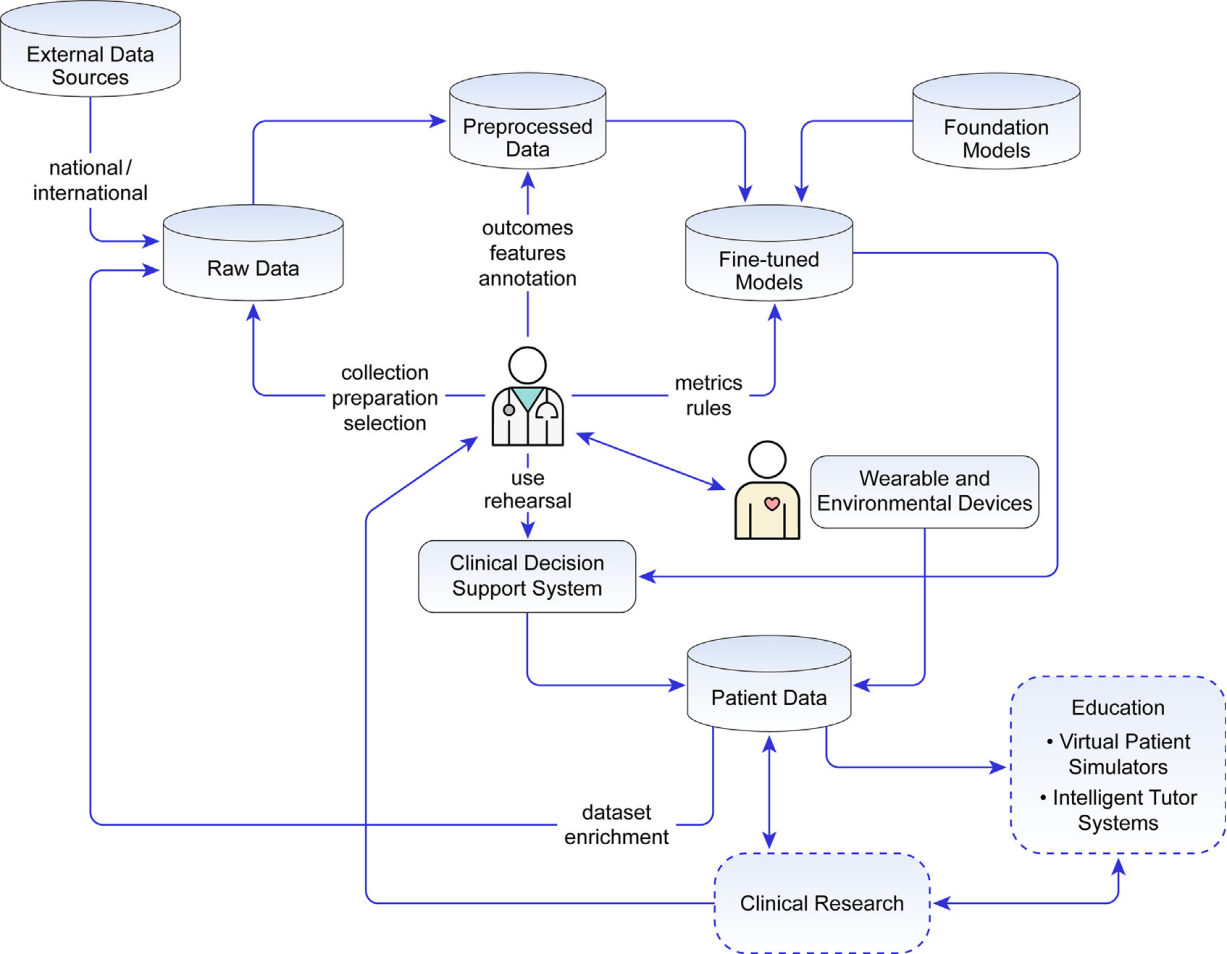
**AI-Driven Technology Gives Encouraging Hope to Improve Syncope Management, as Long as Human Physicians Remain “in the Loop”** Hopes of a novel AI-based versus a standard human-based approach to management of syncope are shown. AI may improve medical education and interactions between clinicians and the patient, as well as more effectively integrate patient-related data and the composite of knowledge on syncope. Therefore, AI has the potential to overcome current impasses inherent in present syncope management. As long as AI technologies keep humans “in the loop,” future aiAI-based clinical decision support systems, research, and medical training may lead to better outcomes. AI = artificial intelligence.

We are in a new era of syncope management in which AI can potentially enhance the clinician’s ability to diagnose and manage patients (Figure 1). It may provide new avenues for syncope research. Our learners are facing new and exciting AI-based educational platforms. Our syncope patients are being empowered by portable AI-powered wearable devices. The complexity of the problem involves nuances in understanding the clinical presentation and mechanisms responsible. While AI in syncope management is in its early stages, its potential is immense. This is particularly important since present management strategies using a standard, non-AI, approach have reached an impasse. In the end, AI has the capability, not yet fully developed, to be better than humans in the overall management of syncope (in collaboration with the clinician), to undertake novel clinical research, and to properly educate trainees perplexed by the complexity of circumstances and patients experiencing syncope-related issues (Figure 1).

## Funding support and author disclosures

The authors have reported that they have no relationships relevant to the contents of this paper to disclose.
